# Basal-Predominant Right-Ventricular Dysfunction in Pediatric Dilated Cardiomyopathy: An Integrated Biventricular Strain Analysis

**DOI:** 10.3390/biomedicines14010038

**Published:** 2025-12-23

**Authors:** Iolanda Muntean, Diana Ramona Iurian, Asmaa-Carla Hagau, Beatrix-Julia Hack

**Affiliations:** 1 Department of Pediatrics III, George Emil Palade University of Medicine, Pharmacy, Science, and Technology of Târgu Mureș, 540136 Targu Mures, Romania; 2 Clinic of Pediatric Cardiology, Emergency Institute for Cardiovascular Diseases and Transplantation of Targu Mureș, 540139 Targu Mures, Romania; 3 Doctoral School of Medicine and Pharmacy, George Emil Palade University of Medicine, Pharmacy, Science, and Technology of Targu Mures, 540136 Targu Mures, Romania; 4 Pediatric Clinical Hospital, 550106 Sibiu, Romania

**Keywords:** pediatric dilated cardiomyopathy, right ventricular strain, right ventricular involvement

## Abstract

**Background:** Right-ventricular (RV) involvement is starting to gain recognition in pediatric dilated cardiomyopathy (DCM), but its deformation characteristics and its relationship to functional status remain insufficiently defined. **Methods:** Twenty-nine children with primary DCM were compared with age- and sex-matched healthy controls. Conventional echocardiography and two-dimensional speckle-tracking echocardiography (2D-STE) were performed. Segmental deformation (basal, mid-ventricular and apical levels) was analyzed using a linear mixed-effects model. Associations between strain indices and advanced functional limitation (NYHA/Ross Class III-IV) were evaluated using exploratory logistic regression and ROC analysis. **Results:** Children with DCM showed significant impairment in both ventricles. Conventional parameters (TAPSE, tricuspid E/A ratio, RV E′ velocity, and LV ejection fraction) were reduced. Right ventricular strain was significantly lower in DCM compared with controls (*p* < 0.05). Segmental analysis demonstrated a basal-predominant reduction in RV strain. Right-ventricular free-wall longitudinal strain correlated with RV S′ (r = −0.49), RV E′ (r = −0.46), LVGLS (r = 0.79) and LV ejection fraction (r = −0.63, all *p* < 0.05). In univariable analysis, RVFWSL predicted advanced functional class (OR 1.13 per 1% decrease, *p* = 0.026), while LVGLS remained the independent predictor in the multivariable model. A combined model incorporating RVFWSL and LVGLS demonstrated excellent discriminative accuracy (AUC 0.95). **Conclusions:** Pediatric DCM is characterized by RV involvement with a distinct basal-dominant deformation pattern. Biventricular strain assessment improves the identification of children with advanced functional class and may enhance functional stratification beyond conventional indices.

## 1. Introduction

The right ventricle (RV) has some unique structural features compared to the left ventricle (LV). Anatomically, the RV contains multiple papillary muscles, a prominent moderator band, and the tricuspid valve, which has an apically displaced septal leaflet. Additionally, it has a completely muscular outflow tract, unlike the fibrous aorto-mitral continuity seen in the LV. In contrast to the LV, which has a well-organized trilaminar myocardial layer, the RV myocardium consists predominantly of a superficial circumferential layer and a deeper subendocardial longitudinal layer [1]. Hemodynamically, the RV is the dominant chamber during fetal life. However, postnatally, after pulmonary vascular resistance declines, the RV walls become thinner and more compliant, whereas the LV adapts to sustain the systemic afterload [2]. Therefore, in a normal heart, the LV contracts mainly through longitudinal and circumferential shortening, while the RV relies on the shortening of longitudinal fibers and motion of the annulus.

Dilated cardiomyopathy was traditionally considered a disease primarily affecting the LV muscle, characterized by LV dilation and impaired systolic function [3]. However, recent studies have shown that the RV is also involved in this condition, with RV dysfunction occurring in 20 to 65% of cases, which has significant prognostic and clinical implications [4,5,6].

Different, but interrelated pathophysiological mechanisms may cause RV dysfunction in idiopathic DCM patients. The first proposed mechanism involves secondary RV involvement caused by the LV dilatation and systolic impairment, which are the hallmarks of DCM. Progressive LV remodeling leads to mitral annular dilatation and functional mitral regurgitation, which can cause post-capillary pulmonary hypertension (Type 2) with RV secondary dysfunction induced by pressure overload [7]. A second mechanism suggests that RV dysfunction may be related to primary cardiomyopathic involvement. For example, recent genetic and imaging studies have demonstrated that different genetic forms of DCM, particularly those involving pathogenic variants in desmosomal genes, desmin, filamin C or Laminin A/C, can produce an overlapping phenotype between DCM and arrhythmogenic cardiomyopathy, often with a biventricular involvement. In these cases, RV dysfunction may occur regardless of LV systolic failure or emerge progressively as part of the evolving cardiomyopathic process, irrespective of the loading conditions [8]. A third mechanism involves the disruption of the ventricular interdependence and LV-RV mechanical coupling. Because the ventricles share the interventricular septum and the pericardial space, LV dilatation and impaired contractility lead to altered septal geometry, annular tethering and loss of basal longitudinal shortening, which compromise RV free-wall deformation and contractility. This geometric remodeling leads to lower mechanical efficiency, with a predisposition to progressive RV dilatation secondary to tricuspid regurgitation that further amplifies RV volume overload [9]. Collectively, these mechanisms indicate that RV involvement in DCM reflects both secondary mechanical and hemodynamic consequences of LV failure, as well as, in some cases, primary or genetic ventricular disease. Furthermore, compared with patients with ischemic DCM, idiopathic DCM patients exhibit a more pronounced RV dysfunction, independent of age, LV function, ventricular size or the presence of pulmonary hypertension, supporting the concept that idiopathic DCM may be characterized by true biventricular involvement [10]. Therefore, recognition of these distinct pathways is crucial for accurate phenotyping, risk stratification and the development of targeted therapeutic strategies in DCM.

The assessment of the RV through imaging techniques varies. Currently, conventional echocardiography remains the most accessible method because it allows for rapid and non-invasive evaluation. The most commonly used parameters in clinical practice include tricuspid annular plane systolic excursion (TAPSE), RV fractional area change (FAC), and tricuspid lateral annular systolic velocity (S′). However, obtaining precise images via echocardiography is limited by the complex shape and position of the RV [11]. Additionally, conventional indices have some limitations: TAPSE only evaluates the basal longitudinal motion of the RV, which can underestimate or overestimate RV dysfunction; RV FAC is difficult to measure accurately because of the RV’s trabeculation that can hide the endocardial tracing; and S’ velocity depends on the angle and loading conditions [6]. Consequently, recent imaging advancements, especially two-dimensional speckle-tracking echocardiography (2D-STE), have enhanced the characterization of RV function. Right ventricle strain analysis can identify subtle systolic abnormalities even when standard indices appear normal [12,13]. Nonetheless, it is infrequently utilized in clinical practice [11]. For quantifying the RV ejection fraction, cardiac magnetic resonance (CMR) imaging is regarded as the gold standard owing to its superior spatial resolution and reproducibility. In pediatric patients with DCM, evaluating RV performance is particularly challenging. Standard echocardiographic parameters may not detect early or regional dysfunction, and CMR is often impractical in children because of the requirement for sedation and limited availability [14,15]. Therefore, STE may provide a more sensitive and reproducible technique for assessing myocardial deformation, enabling simultaneous evaluation of both ventricles [15,16]. However, previous research has primarily focused on left ventricular (LV) strain in children, demonstrating a strong correlation with functional indices and biomarkers that indicate disease severity. Despite the growing use of STE in pediatric echocardiography, segmental RV deformation patterns in pediatric DCM remain scarcely characterized. In particular, there are limited data on RV strain gradients and their relationship with LV mechanics and functional status in children.

Building on our earlier LV-focused work, this study aimed to perform an integrated biventricular analysis of systolic deformation using STE [17]. Specifically, we compared segmental and global strain patterns between the RV and LV, examined the interdependence of their functional parameters, and investigated the predictive value of RV free-wall longitudinal strain (RVFWSL) for clinical severity, as reflected by NYHA functional class. To the best of our knowledge, while RV dysfunction has been previously described in pediatric DCM using conventional echocardiography, tissue-Doppler indices (TDI) or global strain indices, no prior pediatric study has systematically evaluated segmental RVFWSL patterns using two-dimensional STE, nor examined their relationship with LV deformation and functional severity [18,19].

## 2. Materials and Methods

This study was an observational case–control study conducted at the Pediatric Cardiology Clinic of the Emergency Institute for Cardiovascular Diseases and Transplantation in Târgu Mureș, Romania, from January 2022 to December 2023. The study protocol was thoroughly explained to the parents or legal guardians of all participants, and written informed consent was obtained for participation and the anonymous publication of clinical data. The investigation complied with the principles outlined in the Declaration of Helsinki and received approval from the institutional Ethics Committee (approval no. 566/20 January 2022). This analysis extends our previously published cohort, which focused on LV strain assessment [17]. In this expanded study, we evaluated RVFWSL and examined its interaction with LV deformation indices to assess their combined predictive value for clinical severity in pediatric DCM.

### 2.1. Study Population

The study included 29 pediatric patients of Caucasian ethnicity, aged between 1 month and 18 years, diagnosed with primary DCM and followed at our Institution. As recommended in the current guideline, the diagnosis of DCM was established by echocardiography with an increased LV end-diastolic diameter (LVEDD) with a z-score ≥ 2 standard deviations (SD) associated with LV systolic dysfunction [3]. Inclusion criteria comprised age under 18 years and a diagnosis of primary DCM confirmed by echocardiography after excluding secondary causes. Exclusion criteria included age over 18 years and secondary forms of DCM due to hypertension, congenital heart disease, coronary artery anomalies, valvular lesions, neuromuscular, metabolic, infectious, or systemic disorders.

For comparison, 26 healthy children without a history of cardiovascular disease were included as controls, matched for age, sex, anthropometric characteristics, residential background, and ethnicity (Control group).

All participants underwent detailed clinical assessment and comprehensive transthoracic echocardiographic evaluation. A pediatric cardiologist performed a complete physical examination, recording the vital signs and anthropometric measurements. The severity of heart failure (HF) was graded using the pediatric clinical scoring system: the Ross Classification for patients < 5 years of age and the New York Heart Association (NYHA) functional classification for older patients [20,21]. For statistical analyses, functional class was divided into mild-to-moderate (NYHA/Ross I–II) and advanced functional class (NYHA/Ross III–IV). Because this study represents an extension of our previously published LV strain analysis in the same cohort, baseline demographic and clinical characteristics are summarized narratively to avoid duplication of previously published tables.

### 2.2. Conventional Echocardiography

Standard transthoracic echocardiography was performed in all participants using a Philips EPIQ 7 ultrasound system (Philips Medical Systems, Andover, MA, USA) with transducers appropriate for body size. Image acquisition and measurements followed the pediatric recommendations for chamber quantification and functional assessment [14].

Standard two-dimensional, M-mode and Doppler evaluations were used to assess the global systolic and diastolic function of both ventricles. Left ventricular ejection fraction (LV EF) was calculated using the Simpson’s biplane method, and LV end-diastolic dimension (LVEDD) was indexed to body surface area (BSA) and converted to z-scores using pediatric reference data [22]. The RV systolic function was evaluated by TAPSE obtained by M-mode, as well as TDI of the tricuspid annulus: peak systolic velocity (S′). Diastolic function was further assessed from tricuspid flow Doppler by the E/A ratio and early diastolic (E′) and late diastolic (A′) velocities. All measurements were averaged from three cardiac cycles in sinus rhythm.

### 2.3. Speckle-Tracking Analysis

Right and left-ventricular strain was assessed by STE using apical four-chamber (A4C), three-chamber (A3C) and two-chamber (A2C) views, acquired at frame rates between 60 and 90 frames per second. The analysis was performed offline using QLAB 15.5, which incorporated vendor-independent software (TomTec Imaging Systems, Unterschleißheim, Germany). The software automatically detected the endocardial border of each ventricle and was manually adjusted to include the full myocardial wall thickness. Segments with inadequate tracking were manually corrected.

**Figure 1:**
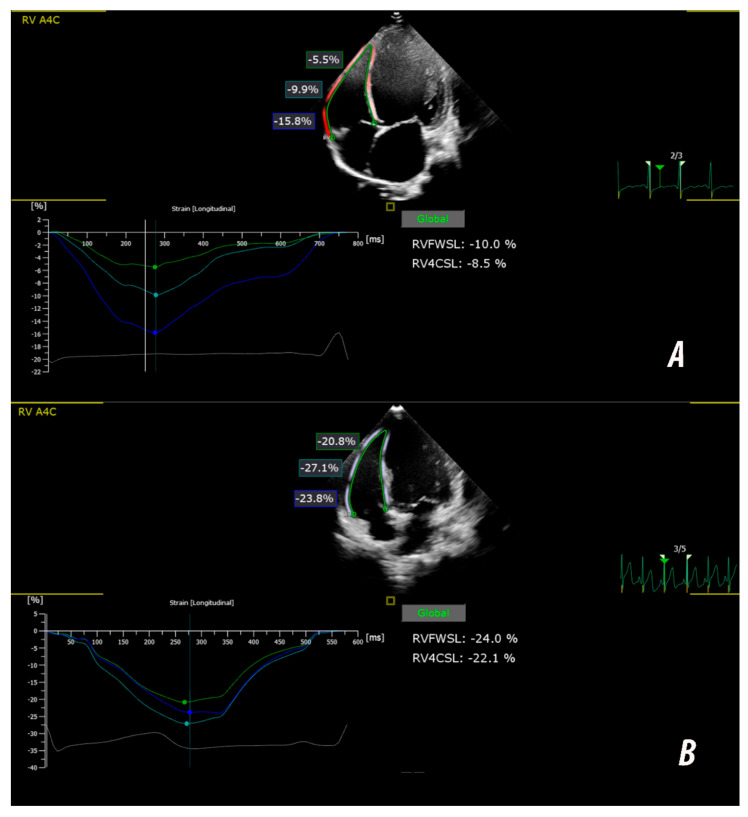
Representative examples of RV STE analysis in the apical four-chamber view. (**A**) Patient with DCM showing severely reduced right-ventricular deformation (RV free-wall longitudinal strain [RVFWSL] −10.0%, RV four-chamber strain [RV4CSL] −8.5%). (**B**) Age-matched healthy control with preserved right-ventricular deformation (RVFWSL −24.0%, RV4CSL −22.1%). Colored curves represent segmental strain profiles for basal, mid-ventricular, and apical free-wall segments, with the average global strain shown in white.

For the RV, images were acquired in an RV-focused A4C, as recommended in the current guidelines [13]. The endocardial borders of the RV free-wall and septum were tracked to derive both global RVFWSL and global four-chamber strain (RV4CSL) (Figure 1). Right-ventricle FWSL was calculated as the average peak systolic strain of the basal, mid and apical segments of the RV free-wall, while RV4CSL included both the septal and the free-wall segments. For the segmental analysis, each of the three RV free-wall segments—basal, mid-ventricular and apical—was evaluated individually, allowing comparison of regional deformation patterns across the RV inflow–mid–apical axis

For the LV, global longitudinal strain (LVGLS) was determined as the average peak systolic strain of 18 segments, according to the current strain analysis recommendation [13]. For the evaluation of regional LV deformation, the 18 longitudinal strain segments were grouped into three anatomical levels- basal, mid-ventricular and apical, each level containing six segments. For each level, segmental peak systolic strain values were averaged to obtain basal, mid-ventricular and apical longitudinal strain indices.

### 2.4. Statistical Tests

Statistical tests were performed using GraphPad Prism version 10 (GraphPad Software, San Diego, CA, USA), SPSS version 29 (IBM Corp., Armonk, NY, USA) and Microsoft Excel 2010. A two-tailed *p*-value < 0.05 was considered statistically significant. The distribution of continuous variables was assessed using the Shapiro–Wilk test. Normally distributed data were presented as mean ± standard deviation (SD), while non-normally distributed variables were expressed as median and interquartile range (IQR). Group comparison between DCM patients and healthy controls was performed using the independent samples *t*-test or Mann–Whitney U test, as appropriate. Categorial variables were compared with the χ^2^ test or Fisher’s exact test. Correlations between continuous variables were assessed using Pearson or Spearman coefficients, depending on data distribution.

In accordance with current recommendations, RVFWSL was used for the assessment and prediction of disease severity [13]. Segmental longitudinal strain values from basal, mid-ventricular, and apical levels of both ventricles were analyzed using a linear mixed-effects model to account for within-subject correlations. The model included ventricle (LV, RV) and segment (basal, mid, apical) as fixed categorical factors, and their interaction (ventricle × segment) to test for regional differences in deformation patterns. Age, body surface area (BSA), and heart rate (HR) were included as covariates because myocardial strain parameters are known to vary with somatic growth, cardiac size and loading conditions in children. Therefore, adjustment for these factors reduces physiological confounding when comparing ventricular deformation patterns. A random intercept-only model was selected to account for inter-individual variability in myocardial deformation. Random slopes were not included because of the limited sample size and the relatively small number of repeated measurements per subject, which could lead to model overfitting and unstable variance estimates. Fixed effects were estimated using restricted maximum likelihood. Pairwise comparisons were adjusted using Bonferroni correction, and estimated marginal means (±95% CI) were plotted to illustrate interaction effects. To explore the relationship between myocardial deformation and functional severity, univariable and multivariable logistic regression analyses were performed with NYHA class (III-IV) as the dependent variable. Given the limited number of outcome events, the multivariable logistic model was specified a priori with two predictors (LVGLS and RVFWSL) and should be interpreted as exploratory. Model calibration was verified using the Hosmer–Lemeshow test, and overall discriminative ability was assessed with receiver-operating-characteristic (ROC) curve analysis. Logistic regression and ROC analysis were performed in the 29 DCM patients (n = 29). The area under the curve (AUC) with 95% confidence intervals was calculated to quantify the model’s accuracy in identifying patients with advanced functional class.

## 3. Results

### 3.1. Baseline Characteristics of the Patients

Baseline demographic and clinical characteristics of the study cohort have been previously reported in our initial analysis focusing on LV deformation and are summarized here to provide context for the extended RV analysis [17].

The DCM group consisted predominantly of male patients (72%), with a mean age of 10.5 ± 6.7 years, compared with 13.5 ± 4.2 years in the control group. Anthropometric measurements followed a similar pattern between groups, although children with DCM tended to have lower body weight (44.3 ± 26.5 kg vs. 52.7 ± 16.4 kg) and height (137.6 ± 47.7 cm vs. 158.8 ± 21.7 cm). Body mass index (BMI) values were comparable (18.2 ± 4.5 kg/m^2^ vs. 20.3 ± 2.9 kg/m^2^), as were the median body surface area (BSA) (1.56 m^2^ [0.70–1.73] vs. 1.44 m^2^ [0.96–1.72]).

Heart-failure severity in the DCM cohort ranged from mild to advanced, with 17% classified as Ross/NYHA I, 27.5% as Class II, 34.4% as Class III, and 20.6% as Class IV. As expected, the DCM group demonstrated a hemodynamic profile consistent with pediatric systolic dysfunction: systolic blood pressure was modestly lower compared with controls (102.9 ± 11.7 mmHg vs. 107.9 ± 9.5 mmHg), and diastolic pressure showed a similar pattern (60.2 ± 11.0 mmHg vs. 70.4 ± 9.6 mmHg). Corresponding percentiles mirrored these differences. Children with DCM also had a higher resting heart rate (87.5 ± 17.0 bpm vs. 79.6 ± 13.6 bpm), reflecting autonomic activation associated with impaired cardiac output. All other demographic indicators were broadly similar, supporting the comparability of groups aside from disease-related physiologic differences. As expected in a tertiary-care DCM population, most patients were receiving guideline-directed medical therapy. Beta-blockers were prescribed in 86.2% (n = 25), ACE inhibitors in 96.6% (n = 28), and diuretics in 93.1% (n = 27). Calcium-channel blockers were not used, and inotropic support had been required in 34.5% (n = 10) during the course of management.

### 3.2. Conventional Echocardiographic Indices

Compared with healthy controls, patients with DCM exhibited significantly lower indices of both LV and RV systolic function (Table 1).

Tricuspid annular plane systolic excursion (TAPSE) was reduced in DCM (1.96 ± 0.48 cm) compared to controls (2.29 ± 0.47 cm, *p* < 0.01), indicating early RV systolic impairment. Tricuspid inflow analysis revealed a significantly lower E/A ratio in DCM compared to controls (1.26 ± 0.35 vs. 1.55 ± 0.18, *p* < 0.01), consistent with impaired RV diastolic relaxation.

Tissue-Doppler imaging demonstrated markedly decreased RV E′ velocities (10.97 ± 3.23 cm/s vs. 15.00 ± 2.18 cm/s, *p* < 0.01), while RV S′ showed a non-significant downward trend (*p* = 0.06). No differences were observed for RV A′ velocities.

Left-ventricular systolic performance was severely reduced in DCM, with significantly lower LVEF (42.6 ± 17.9% vs. 73.06 ± 5.93%, *p* < 0.01).

Together, these data confirm combined RV and LV systolic dysfunction in pediatric DCM, with evidence of early RV diastolic abnormalities.

### 3.3. Right-Ventricular Deformation Analysis

Right-ventricular (RV) deformation was significantly reduced in patients with DCM compared with healthy controls.

**Figure 2:**
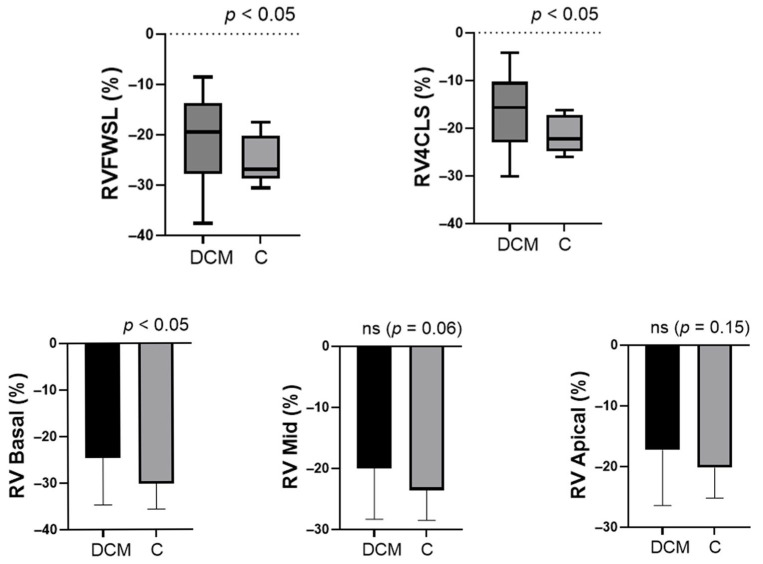
Comparison of right-ventricular longitudinal strain parameters between pediatric patients with DCM and healthy controls. (**Top**) Global right-ventricular free-wall longitudinal strain (RVFWSL) and right-ventricular four-chamber strain (RV4CSL) were significantly reduced in the DCM group compared with controls (*p* < 0.05). (**Bottom**) Segmental analysis revealed that basal RV strain was significantly decreased in DCM (*p* < 0.05), whereas mid-ventricular (*p* = 0.06) and apical (*p* = 0.15) segments showed non-significant differences (ns). Data are expressed as mean ± SD or median [IQR], according to distribution. Group comparisons were performed using the independent-samples *t*-test or Mann–Whitney U test, as appropriate. Sample size: DCM, n = 29; Controls, n = 26.

Median global RVFWSL was significantly lower in the DCM group (−19.5%) than in controls (−26.9%; *p* < 0.05). Segmental analysis showed significantly reduced strain in the basal RV segment (−24.6 ± 10.1% vs. −30.2 ± 5.44%, *p* < 0.05), a trend toward reduction in the mid-segment (−19.99 ± 8.34% vs. −23.60 ± 4.91%, *p* = 0.06) and no significant difference in the apical segment (−17.31 ± 9.12% vs. −20.25 ± 4.96%, *p* = 0.150) (Figure 2). Although the apical segment showed the lowest absolute strain values, the most pronounced relative reduction compared with controls occurred in the basal region.

Global RV strain measured in the four-chamber view was also significantly reduced in DCM (−15.7%) compared with controls (median −22.3%; *p* <0.01). Collectively, these results demonstrate a pattern of basal-predominant RV dysfunction and overall reduction in global longitudinal strain in pediatric DCM, with relative preservation of apical deformation.

### 3.4. Correlation Analysis

**Figure 3:**
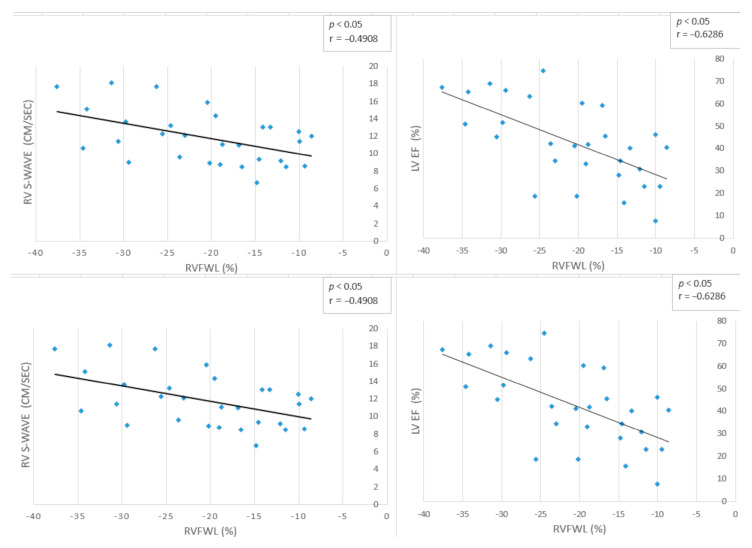
Correlations between right-ventricular free-wall longitudinal strain and echocardiographic functional indices in pediatric dilated cardiomyopathy (DCM). RVFWSL showed significant negative correlations with RV systolic velocity (RV S′; r = –0.49, *p* < 0.05), RV early diastolic velocity (RV E′; r = –0.46, *p* < 0.05), and LV ejection fraction (LV EF; r = –0.63, *p* < 0.05). A strong positive correlation was observed between RVFWSL and LV global longitudinal strain (LVGLS; r = 0.79, *p* < 0.05). Correlation analyses were performed in the DCM group (n = 29).

In the DCM group, RVFWSL correlated significantly with both right- and left-sided functional indices (Figure 3). Reduced RV deformation was associated with lower RV systolic and diastolic velocities, specifically with RV S′ (r = −0.49, *p* < 0.05) and RV E′ (r = −0.46, *p* < 0.05), as well as with decreased early tricuspid inflow velocity E (r = −0.43, *p* < 0.05). Right ventricular free-wall strain also correlated strongly with LV systolic parameters, showing a negative association with LV EF (r = −0.63, *p* < 0.05) and a positive association with LVGLS (r = 0.79, *p* < 0.05). Structural remodeling was reflected by positive correlations between RVFWSL and both LVEDD (r = 0.41, *p* < 0.05) and LVEDD Z-score (r = 0.48, *p* < 0.05). No significant association was observed with tricuspid E/A ratio or RV A′ velocity. These findings highlight a close mechanical interdependence between the two ventricles, demonstrating that RV deformation worsens in parallel with both systolic impairment and ventricular dilation in pediatric DCM.

### 3.5. Differential Segmental Remodeling Patterns in the Ventricles

**Figure 4:**
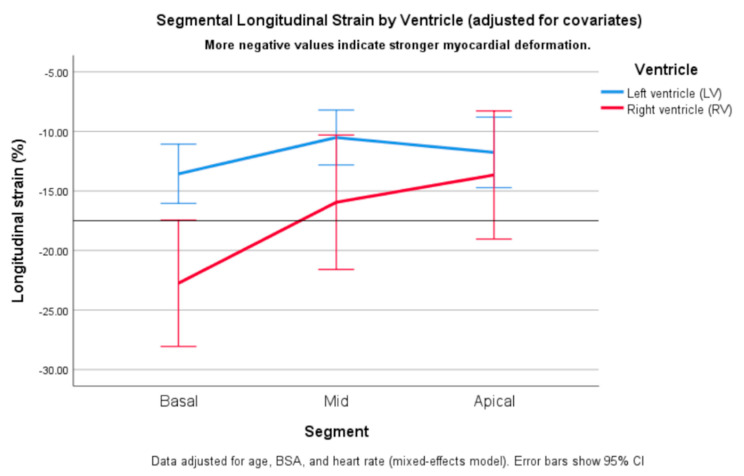
Segmental longitudinal strain by ventricle (adjusted for age, body surface area, and heart rate). Estimated marginal means of longitudinal strain (±95% CI) are shown for left (LV, blue) and right (RV, red) ventricles across basal, mid-ventricular, and apical segments. More negative values indicate greater myocardial deformation. RV strain was significantly more negative than LV strain at the basal (*p* < 0.001) and mid-ventricular (*p* = 0.009) levels, whereas apical values did not differ significantly (*p* = 0.23). Both ventricles demonstrated a basal-to-apical decline in deformation magnitude. Analyses were performed in the DCM cohort (n = 29).

In the mixed-effects model adjusted for age, BSA, and HR, both ventricular chamber (LV vs. RV) and myocardial segment (basal, mid-ventricular, apical) had significant effects on longitudinal strain (*p* < 0.01 for both main factors), with a significant ventriculo-segmental interaction (*p* < 0.01). Overall, RV strain was more negative than LV longitudinal strain. Pairwise comparisons showed the greatest LV–RV difference at the basal level (Δ ≈ −11%; *p* < 0.01), a smaller but still significant difference at the mid-ventricular level (Δ ≈ −7%; *p* < 0.05), and no significant difference at the apical level (Figure 4). The estimated marginal means and corresponding 95% CI derived from the mixed-effects model are provided in Supplementary Table S1.

This pattern suggests a basal-to-apical gradient of LV–RV deformation, reflecting mechanical coupling between the ventricles. With disease progression, LV dilation and reduced septal motion likely impair basal shortening, while apical regions remain relatively preserved.

### 3.6. Predictive Models of Functional Severity

In the univariable logistic regression model, RVFWSL was significantly associated with functional NYHA class. Each 1% decrease in RV systolic deformation (less negative strain) increased the odds of being in advanced functional class (NYHA III–IV) by 13% (B = 0.124, OR = 1.13, 95% CI 1.02–1.26, *p* = 0.026). Given the limited number of patients in advanced NYHA/Ross class, the multivariable logistic regression model should be interpreted as exploratory. Collinearity diagnostics were assessed to evaluate potential multicollinearity between LVGLS and RVFWSL. Variance inflation factor (VIF) values were <2 for both predictors, indicating no relevant multicollinearity.

However, to test the independent predictive value of RVFWSL, we inserted into the multivariable model both RVFWSL and LVGLS simultaneously, without stepwise selection, as predictors of functional severity (NYHA class III–IV). The overall model was highly significant (χ^2^ = 26.8, df = 2, *p* < 0.001) and demonstrated excellent calibration (Hosmer–Lemeshow *p* = 0.99). Left ventricular GLS emerged as an independent predictor of severe functional limitation (B = 1.33 ± 0.64, *p* = 0.039, OR = 3.77 [95% CI 1.07–13.26]), whereas RVFWSL showed a borderline inverse association (B = −0.57 ± 0.34, *p* = 0.095, OR = 0.56 [0.29–1.11]). These findings indicate that both LV and RV systolic deformation relate closely to functional limitation, but with LVGLS exerting the stronger independent effect. The attenuation and change in the direction of the RVFWSL coefficient in the multivariable model likely reflect shared variance with LVGLS and the strong mechanical coupling between the two ventricles, rather than true statistical multicollinearity.

**Figure 5:**
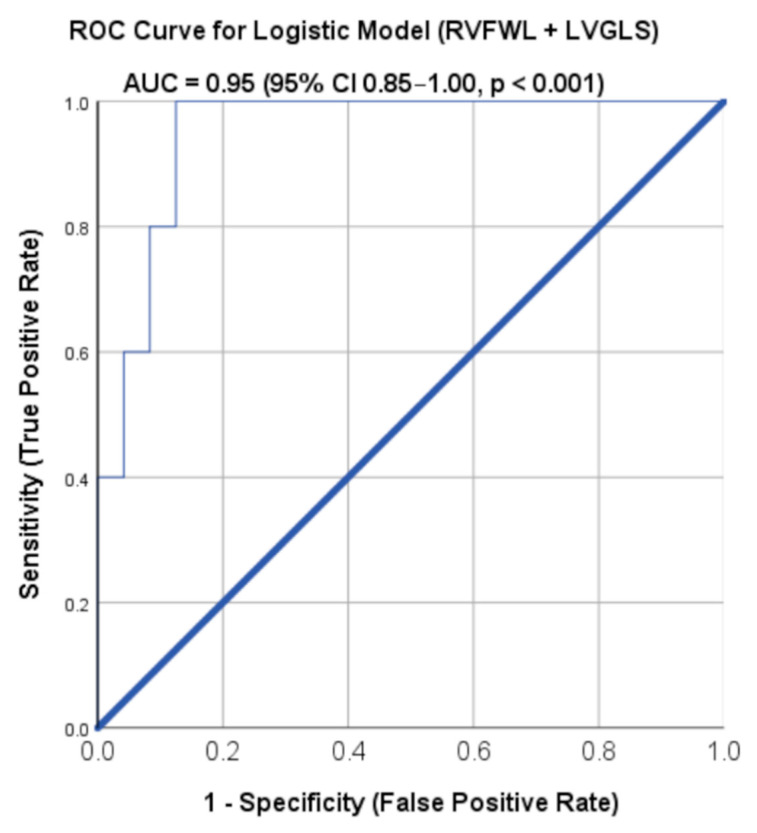
Receiver-operating characteristic (ROC) curve for the logistic regression model combining right-ventricular free-wall longitudinal strain (RVFWSL) and left-ventricular global longitudinal strain (LVGLS) in predicting severe functional limitation (NYHA/Ross class III–IV) among pediatric patients with dilated cardiomyopathy. The model demonstrated excellent discriminative ability, with an area under the curve (AUC) of 0.95 (95% CI 0.85–1.00, *p* < 0.001), indicating high accuracy for distinguishing between mild-to-moderate (NYHA/Ross I–II) and advanced functional class (NYHA/Ross III–IV) disease. Analyses were performed in the DCM cohort (n = 29).

Receiver-operating-characteristic (ROC) analysis confirmed excellent discriminative capacity for the combined strain model (AUC = 0.95, 95% CI 0.85–1, *p* < 0.01). A predicted probability threshold of 0.06 achieved 100% sensitivity and 87.5% specificity for identifying patients with advanced functional class (NYHA/Ross III–IV) (Figure 5). The optimal probability threshold (0.06) determined using the Youden index was selected to prioritize sensitivity for identifying patients with advanced functional limitation, which is clinically relevant in pediatric DCM, where early recognition of severe disease is essential. These results indicate that impaired longitudinal deformation strongly associates with clinical severity, while RV strain contributes additional explanatory value.

## 4. Discussion

This study extends our previous research, which focused solely on LV strain analysis in the same pediatric DCM cohort, by providing a comprehensive evaluation of RV deformation using STE [17]. While prior pediatric studies have predominantly focused on global RV functional indices or TDI parameters, detailed assessment of regional RV deformation has remained limited [18,19]. Therefore, by including RVFWSL and direct biventricular comparison, we aimed to highlight interventricular deformation patterns and their relationship with clinical severity.

Our findings demonstrate that RV dysfunction is a frequent and clinically relevant feature of pediatric DCM, closely associated with LV systolic impairment and functional limitation. To our knowledge, this is the first systematic evaluation of segmental RVFWSL in pediatric DCM, demonstrating a reproducible basal-predominant strain pattern with relative apical preservation.

Consistent with earlier pediatric reports, our cohort showed significant LV systolic dysfunction and chamber dilation, with RV impairment [18,23]. Reduced TAPSE, lower tricuspid E/A ratio, and decreased RV TDI indicated early RV systolic and diastolic involvement. The strong correlations between RVFWSL and both LVGLS and LV EF highlight the close mechanical interdependence between the ventricles, where LV dilation and septal motion abnormalities negatively affect RV deformation. Structural indices such as LVEDD and LVEDD Z-score were also related to RV strain, suggesting that progressive remodeling alters biventricular geometry and contractile synchrony [19].

In this pediatric DCM cohort, we observed distinct spatial deformation patterns in the two ventricles. In line with our previous findings, LV longitudinal strain reduction was most pronounced in the mid-ventricular region, resulting in an attenuated base-to-apex gradient consistent with diffuse LV systolic dysfunction [17]. In contrast, RV analysis revealed predominant basal impairment, with relative preservation of the mid- and apical segments. In contrast, RV analysis revealed predominant basal impairment, with relative preservation of mid- and apical segments. Although absolute strain values were lowest at the apical level in both patients and controls, the disease-related reduction was greatest at the basal RV segment. Thus, the term “basal-predominant impairment” refers to the relative reduction in deformation that is attributed to the disease, rather than to absolute strain magnitude. Preservation of mid- and apical-segment strain suggests early, regionally predominant involvement of the RV inflow region and may precede deterioration of conventional RV functional indices.

The mixed-effects model confirmed significant main effects of both ventricle and segment, as well as a ventriculo-segmental interaction, supporting the concept of different remodeling: diffuse LV involvement with mid-ventricular predominant impairment versus predominant RV basal-dominant dysfunction with relative apical preservation. The relative preservation of apical strain across both ventricles likely reflects shared longitudinal fiber continuation through the interventricular septum and coordinated electromechanical activation [24,25]. This pattern may be due to different wall stress distributions, fiber architecture, and ventricular interdependence [9,25]. In contrast, basal RV basal function is more dependent on annular and septal motion, which may be compromised by LV dilatation and septal tethering, while shared longitudinal fiber continuity helps preserve apical deformation until later disease stages. The absence of statistically significant differences at the mid- and apical levels may also reflect the limited sample size and the inherent variability of regional RV strain measurements in pediatric echocardiography.

Evidence from the adult DCM population is heterogeneous and often inconsistent, likely reflecting differences in methodology, disease stage and imaging techniques. Interestingly, a similar segmental pattern that we found has been described in mutation-positive arrhythmogenic RV cardiomyopathy, where basal involvement precedes apical dysfunction [26]. These findings suggest that RV basal impairment may indicate early functional decline across different cardiomyopathies, regardless of etiology. In contrast, Ali et al. recently reported apical RV impairment in adult DCM using TDI-derived strain [27]. This discrepancy may be explained by methodological limitations of TDI, including angle dependence and lower reproducibility, as well as differences between adult and pediatric disease phenotypes [28]. Moreover, studies in pulmonary hypertension have demonstrated reversal of the physiological RV strain gradient, with the apical strain most depressed and the basal segment relatively preserved [29,30]. Similarly, in systemic sclerosis, complex interactions between pulmonary vascular disease, RV afterload, and intrinsic myocardial involvement drive a characteristic pattern of RV remodeling and dysfunction [31]. These observations underscore that RV deformation patterns are disease-specific and influenced by distinct loading conditions and myocardial architecture.

In the pediatric population, data are still scarce. However, our findings align with prior pediatric echocardiographic studies that emphasize the importance of ventricular interaction in pediatric DCM. For example, Agha et al. demonstrated that both systolic and diastolic RV function are significantly impaired in children with DCM, with strong correlations between RV indices and LVGLS [18]. Similarly, in our cohort, reduced RV deformation closely paralleled LV dysfunction and functional severity. In the univariable logistic model, RVFWSL was significantly associated with advanced functional class, confirming its potential clinical relevance. This observation is broadly consistent with adult data, in which impaired RV strain has been associated with adverse outcomes [5]. However, when combined with LVGLS, LV strain remained the dominant independent determinant of functional severity, whereas RVFWSL showed only a borderline association. These findings suggest that although RV deformation contributes to overall hemodynamic compromise, LV contractile impairment remains the principal driver of symptomatic limitation in pediatric DCM. In our previous analysis, GLS demonstrated strong discriminatory power for the advanced functional class [17]. In the present extended model, incorporating RVFWSL further improved the overall predictive performance. This highlights the complementary value of biventricular strain analysis in pediatric DCM.

From a clinical perspective, early recognition of biventricular dysfunction may support more informed therapeutic decision-making and optimize the timing of advanced interventions. Identification of early basal RV dysfunction may help refine the timing of escalation in children with progressive DCM. Incorporating RV strain into standard echocardiographic protocols may improve the detection of subclinical disease progression and refine functional assessment beyond conventional parameters such as LV EF or TAPSE.

## 5. Limitations

This study has several limitations. First, although our center is the largest pediatric cardiology and heart-transplant facility in our country, being the national reference center for children with DCM, it remains a single tertiary institution. Therefore, the relatively small sample size may limit the statistical power and generalizability of our findings. Second, the cross-sectional design precludes evaluation of longitudinal ventricular remodeling or outcome prediction. Third, RV and LV deformation were assessed by using 2D-STE with a single-vendor and RV analysis based solely on the A4C view, without CMR validation or inter-observer reproducibility testing. Right-ventricular free-wall strain assessment is inherently sensitive to image acquisition and probe positioning, particularly in pediatric patients, and the absence of reproducibility analysis therefore represents a limitation of the present study. However, according to the latest guidelines, the A4C-focused strain analysis of the RV is the recommended approach and, as stated, CMR validation is challenging in pediatrics because of sedation requirements, limited availability and cost [13,15]. In addition, all examinations were performed using the same ultrasound system and vendor-independent software, which reduces methodological variability. Prior comparative studies have shown that TomTec generally provides lower measurement variability and more consistent tracking performance compared to other platforms [32,33]. Fourth, the number of patients in advanced NYHA/Ross functional class was modest, reflecting the rarity of severe pediatric DCM. To minimize model instability, calibration was assessed, and sensitivity checks were performed, although these exploratory models require confirmation in larger, multicenter cohorts. Therefore, additional multicenter studies with larger cohorts, serial follow-up and outcome-based endpoints are required to confirm the prognostic significance of combined LV–RV strain assessment in pediatric DCM.

## 6. Conclusions

Right ventricular deformation is significantly reduced in pediatric DCM and closely interacts with LV dysfunction. A combined model including RVFWSL and LVGLS provides excellent discrimination of clinical severity, supporting the concept of integrated biventricular strain evaluation as a sensitive marker of disease burden and functional limitation in children with DCM.

## Figures and Tables

**Table 1:** Conventional echocardiographic parameters in pediatric patients with dilated cardiomyopathy (DCM) compared with healthy controls.

Variable	DCM (n = 29)	Control (n = 26)	*p*-Value
TAPSE (cm)	1.96 ± 0.48	2.29 ± 0.47	<0.01
E wave (m/s)	0.6 [0.54–0.88]	0.7 [0.62–0.82]	0.1
A wave (m/s)	0.57 ± 0.22	0.47 ± 0.09	0.06
E/A	1.26 ± 0.35	1.55 ± 0.18	<0.01
RV S′ (cm/s)	11.83 ± 3.02	13.15 ± 1.42	0.06
RV E′ (cm/s)	10.97 ± 3.23	15 ± 2.18	<0.01
RV A′ (cm/s)	10.71 ± 3.24	10.43 ± 3.38	0.5
LV EF (%)	42.59 ± 17.9	73.06 ± 5.93	<0.01
LVEDD (cm)	5.61 [3.96–8.45]	4.28 [3.77–4.85]	<0.01
LVEDD Z SCORE	4.29 [2.43–5.38]	−0.9 [−2–0.11]	<0.01

Values are expressed as mean ± standard deviation (SD) for normally distributed variables and median [interquartile range] for non-normally distributed variables. Abbreviations: TAPSE—tricuspid annular plane systolic excursion; E/A—tricuspid inflow ratio; RV S′, E′, A′—tricuspid annular systolic, early, and late diastolic velocities; LVEF—left ventricular ejection fraction; LVEDD—left ventricular end-diastolic diameter; m/s—meters/second; cm/s—centimeters/second.

## Data Availability

The data presented in this study are available on reasonable request from the corresponding author. The data are not publicly available due to ethical restrictions related to patient confidentiality and the inclusion of pediatric participants.

## Supplementary Materials

The following supporting information can be downloaded at: https://www.mdpi.com/article/10.3390/biomedicines14010038/s1, Table S1. Estimated marginal means (±95% confidence intervals) of segmental longitudinal strain derived from the linear mixed-effects model adjusted for age, body surface area, and heart rate.

## Abbreviations

The following abbreviations are used in this manuscript:

RV	Right ventricle/right ventricular
LV	Left ventricle/left ventricular
DCM	Dilated cardiomyopathy
RVFWSL	Right ventricular free-wall longitudinal strain
RV4CSL	Right-ventricular four-chamber longitudinal strain
LVGLS	Left-ventricular global longitudinal strain
LVEF	Left ventricular ejection fraction
LVEDD	Left-ventricular end-diastolic diameter
LVEDD z-score	Left ventricular end-diastolic diameter z-score
TAPSE	Tricuspid annular plane systolic excursion
FAC	Fractional area change (right ventricle)
S′	Peak systolic tissue Doppler velocity of the tricuspid annulus
E′	Early diastolic tissue Doppler velocity of the tricuspid annulus
A′	Late diastolic tissue Doppler velocity of the tricuspid annulus
E/A	Early-to-late diastolic transtricuspid inflow velocity ratio
HF	Heart failure
NYHA	New York Heart Association (functional classification)
BSA	Body surface area
BMI	Body mass index
SD	Standard deviation
IQR	Interquartile range
AUC	Area under receiver-operating-characteristic curve
CI	Confidence interval
HR	Heart rate
TDI	Tissue Doppler imaging
2D-STE	Two-dimensional speckle-tracking echocardiography
CMR	Cardiac magnetic resonance imaging
A4C	Apical four-chamber view
A3C	Apical three-chamber
A2C	Apical two-chamber
ROC	Receiver-operating characteristic (curve)
