# Supplementary material for: Basal-Predominant Right-Ventricular Dysfunction in Pediatric Dilated Cardiomyopathy: An Integrated Biventricular Strain Analysis
Source: Biomedicines. 2025 Dec 23;14(1):38. doi: 10.3390/biomedicines14010038 (PMC12838392; doi:10.3390/biomedicines14010038)
Supplement: Supplementary file 1 [file biomedicines-14-00038-s001.zip › biomedicines-4044624-supplementary.pdf]

Table S1. Estimated marginal means (±95% confidence intervals) of segmental longitudinal strain derived from the linear mixed-effects model adjusted for age, body surface area, and heart rate.

Ventricle	Segment	Estimated marginal mean (%)	95% CI (lower)	95% CI (upper)
LV	Basal	-13.56	-17.58	-9.54
LV	Mid-ventricular	-10.51	-14.82	-6.21
LV	Apical	-11.76	-16.07	-7.45
RV	Basal	-22.76	-26.78	-18.74
RV	Mid-ventricular	-15.95	-20.26	-11.64
RV	Apical	-13.66	-17.97	-9.35
Estimated marginal means were derived from a linear mixed-effects model including ventricle and segment as fixed effects, with age, body surface area, and heart rate as covariates, and a random intercept for subject. Analyses were performed in the DCM cohort (n = 29).				
